# How to improve the structural stabilities of halide perovskite quantum dots: review of various strategies to enhance the structural stabilities of halide perovskite quantum dots

**DOI:** 10.1186/s40580-024-00412-x

**Published:** 2024-01-27

**Authors:** Dokyum Kim, Taesun Yun, Sangmin An, Chang-Lyoul Lee

**Affiliations:** 1 Advanced Photonics Research Institute (APRI), Gwangju Institute of Science and Technology (GIST), Gwangju, 61005 Republic of Korea; 2 Department of Physics, Research Institute of Physics and Chemistry, Jeonbuk National University, Jeonju, 54896 Republic of Korea

**Keywords:** Halide perovskite, Quantum dots, Stability

## Abstract

Halide perovskites have emerged as promising materials for various optoelectronic devices because of their excellent optical and electrical properties. In particular, halide perovskite quantum dots (PQDs) have garnered considerable attention as emissive materials for light-emitting diodes (LEDs) because of their higher color purities and photoluminescence quantum yields compared to conventional inorganic quantum dots (CdSe, ZnSe, ZnS, etc.). However, PQDs exhibit poor structural stabilities in response to external stimuli (moisture, heat, etc*.*) owing to their inherent ionic nature. This review presents recent research trends and insights into improving the structural stabilities of PQDs. In addition, the origins of the poor structural stabilities of PQDs and various methods to overcome this drawback are discussed. The structural degradation of PQDs is mainly caused by two mechanisms: (1) defect formation on the surface of the PQDs by ligand dissociation (*i.e.,* detachment of weakly bound ligands from the surface of PQDs), and (2) vacancy formation by halide migration in the lattices of the PQDs due to the low migration energy of halide ions. The structural stabilities of PQDs can be improved through four methods: (1) ligand modification, (2) core–shell structure, (3) crosslinking, and (4) metal doping, all of which are presented in detail herein. This review provides a comprehensive understanding of the structural stabilities and opto-electrical properties of PQDs and is expected to contribute to future research on improving the device performance of perovskite quantum dot LEDs (PeLEDs).

## Introduction

Displays have become an essential part of society, ranging from portable electronics such as smartphones and tablets to stationary electronics such as monitors and TVs. These displays are utilized for a variety of activities, including watching digital content, writing, and reading documents. Consequently, there is a high demand for displays that can provide a realistic color gamut, low energy consumption, outdoor visibility, and flexibility. These requirements have led to extensive research in the field of displays [1].

In recent years, light emitting diodes (LEDs) have become one of the most actively researched display technologies. LEDs exhibit superior characteristics compared to liquid crystal displays (LCDs) in terms of brightness, contrast ratio, response time, and viewing angle. These LEDs can be categorized into organic LEDs (OLEDs) and quantum dot LEDs (QLEDs) based on the material used as the emissive layer [2]. High brightness displays can be realized using OLEDs because light is emitted directly from pixels without a color filter [3]. Additionally, they can be implemented on flexible displays. However, the poor device performance and short operating lifetime of blue OLEDs are bottlenecks for the realization of full color displays [4]. QLEDs, which utilize inorganic quantum dots (CdSe, ZnSe, ZnS, etc*.*) as emissive materials, can realize a more vivid color with a narrower full width at half maximum (FWHM) than OLEDs. QLEDs also have the advantage of simple thin film fabrication via solution process (spin-coating). Thus, QLEDs have attracted significant attention in recent years [5].

Perovskite LEDs (PeLEDs) using halide perovskite quantum dots (PQDs) as emissive materials have emerged as next generation displays beyond OLEDs and QLEDs. They utilize a quantum confinement effect similar to that in QLEDs, but exhibit superior performance compared to the latter. PeLEDs exhibit high photoluminescence quantum yields (PLQYs, > 80%) and a narrow FWHM (< 20 nm), similar to or better than those of well-known II-IV inorganic QDs, resulting in enhanced color purity compared to QLEDs [6, 7]. In addition, the optical bandgap (electroluminescence (EL) and photoluminescence (PL) emission) of the PQDs can be easily tuned by controlling the halide composition (Cl, Br, and I) to achieve full colors of blue, green, and red. The external quantum efficiency (EQE) of PeLEDs has improved, significantly, achieving 15%, 24.94%, and 23.5% for blue, green, and red, respectively [8–12]. The maximum luminescence of PeLEDs is 8,136 cd/m^2^ for blue, 25,566 cd/m^2^ for green, and 12,910 cd/m^2^ for red, respectively [13–15]. PQDs refer to nanoparticles with sizes of ~ 10–15 nm and the chemical formula ABX_3_. In this chemical structure, A**–**site cations (*e.g.,* Cs^+^, CH_3_NH_3_^+^) occupy the corners of the cubic unit cell, B**–**site cations (*e.g.,* Pb^2+^, Sn^2+^) occupy the body-center, and X**–**site anions (Cl^−^, Br^−^, and I^−^) are located at the face-center. The B**–** and X**–**site ions form an octahedral shape of [BX_6_]^4−^ within the crystal structure [16]. PQDs exhibit excellent optoelectronic properties, comprising the advantages of both perovskites and quantum dots (nanoparticles), which have led to their widespread use in various applications, including solar cells, LEDs, and photodetectors [17–29].

**Scheme 1:**
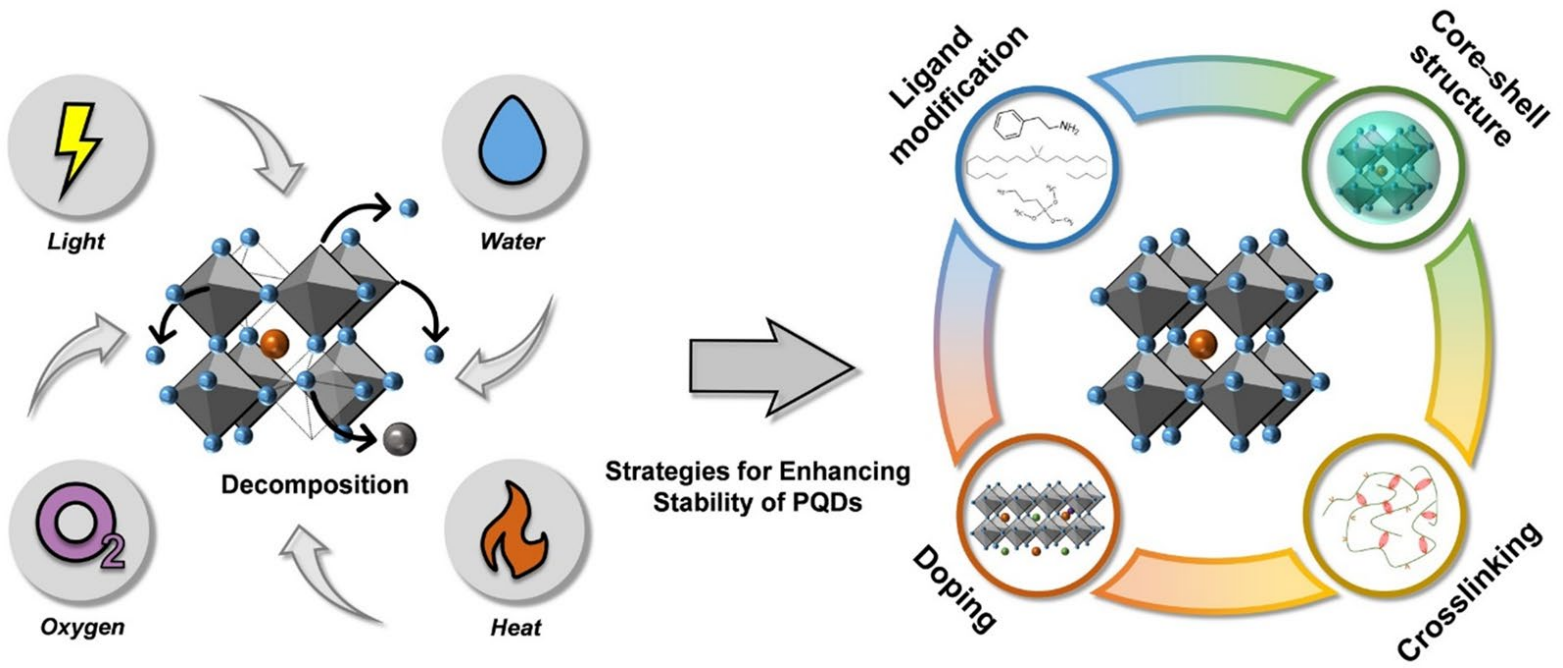
External stimuli that contribute to the structural degradation of PQDs and various strategies to improve their structural stabilities.

However, despite their excellent optoelectronic properties, PQDs have inherently low structural stabilities because of their ionic nature [30, 31]. PQDs are easily decomposed or degraded under external stimuli (moisture, oxygen, and heat, etc.) The structural degradation of PQDs is primarily caused by the formation of defects (vacancies and interstitials) through ion migration and the detachment of weakly bound ligands on the PQD surface [32, 33]. Owing to the relatively low ionic migration energy within the lattices of PQDs, the formation energy of halide vacancies is also low, facilitating the easy formation of defects in the lattice [32, 33]. Consequently, PQDs are easily degraded by external stimuli [34, 35]. Furthermore, weakly bound ligands on the PQD surface are easily detached during purification processes or when exposed to ambient conditions. This leads to the aggregation of PQDs, which accelerates their structural degradation upon exposure to external stimuli [33, 35]. This structural degradation increased non-radiative recombination and reduced the PLQY. Therefore, methods to suppress defect formations and to heal defects both in the lattice (core of PQDs) and on the surfaces are urgently needed. Four strategies have been intensively studied to improve the extrinsic (defect formation on the surface) and intrinsic (defect formation in the lattice) structural stabilities of PQDs; ligand modification, core–shell structure, crosslinking, and metal doping. These strategies will be reviewed in detail in the following section Scheme 1.

## Strategies for improving structural stabilities of PQDs

Various attempts have been made to improve the structural stabilities (extrinsic and intrinsic) of PQDs during the synthesis processes, such as ligand-assisted re-precipitation (LARP) and hot-injection, as well as post-treatment processes after PQDs fabrication [36, 37]. (1) Ligand modification: Long alkyl chain ligands (*e.g.,* oleic acid (OA, C_18_H_34_O_2_) and oleylamine (OAm, C_18_H_37_N_2_)) are exchanged with various ligands to improve ligand packing density and reduce steric hindrance, thereby inhibiting defect formation. (2) Core–shell structure: Polymers or inorganic materials are introduced as passivation layers to improve the structural stabilities of PQDs against external stimuli (moisture or oxygen). (3) Crosslinking: Crosslinkable ligands are introduced as surface passivation ligands to minimize defect formation by inhibiting ligand dissociation through the crosslinking of ligands via light or heat. (4) Metal doping: Metal ions with equivalent charge numbers are doped at the A– or B–sites, which significantly improves the structural stabilities of PQDs by changing the B–X bond lengths. Notably, the Goldschmidt tolerance and octahedral factors should be maintained when doping PQDs with various metal ions. Methods (1)–(3) can be carried out by an *in-situ* process during PQDs synthesis or as post-treatment process after PQDs fabrication; whereas, method (4) is typically done *in-situ* during PQDs synthesis. The following subsections detail the characteristics and applications of each of these methods.

### Ligand modification

During PQDs synthesis, the size and shape of the PQDs can be controlled by changing the type of acid and amine used as ligands [38–40]. When long alkyl chain ligands such as OA and OAm, which have low mobility, are used as surface ligands, PQDs are formed as a zero-dimensional structure owing to slow crystal growth rate [41]. However, OA and OAm have molecular structures in which the middle of the chain is bent by double bonds. This causes steric hindrance, which reduces ligand packing density on the PQD surface. Consequently, structural degradation occurs when the PQD surfaces without passivation are exposed to external stimuli [42]. To address these issues and improve the structural stabilities of PQDs, many studies have focused ligand modification via post-treatment processes [43–47].

After the fabrication of PQDs by either hot-injection or LARP, a purification process with polar solvents such as methyl acetate or butanol is required to remove excess ligands [45] and by-products and obtain a narrow particle size distribution [48]. However, during purification, the ligands bound to the surface of the PQDs are easily detached and undergo structural degradation. Bi et al*.* reported that the surface defects generated during the purification of CsPbI_3_ QDs can be healed through a post-treatment process [49] utilizing the strong affinity between 2-aminoethanethiol (AET) and Pb^2+^. The thiolate groups in AET bind more strongly with the Pb^2+^ on the surface of PQDs compared to OA or OAm, forming a dense barrier (*i.e.* passivation) layer on the surface. This passivation layer effectively prevents structural degradation caused by moisture (water) and UV exposure. Even after 60 min of water exposure or 120 min of UV exposure, the PQDs maintained their cubic phase without any decomposition or phase transition, and their PL intensity remained above 95% of its initial value. The PLQYs improved from 22 to 51% after ligand exchange with AET ligands. In addition to improving the structural stability and PLQY, the shorter inter-particle distance between PQDs, owing to the short alkyl chain length of the AET ligand, also enhanced the photodetector performance.

**Fig. 1:**
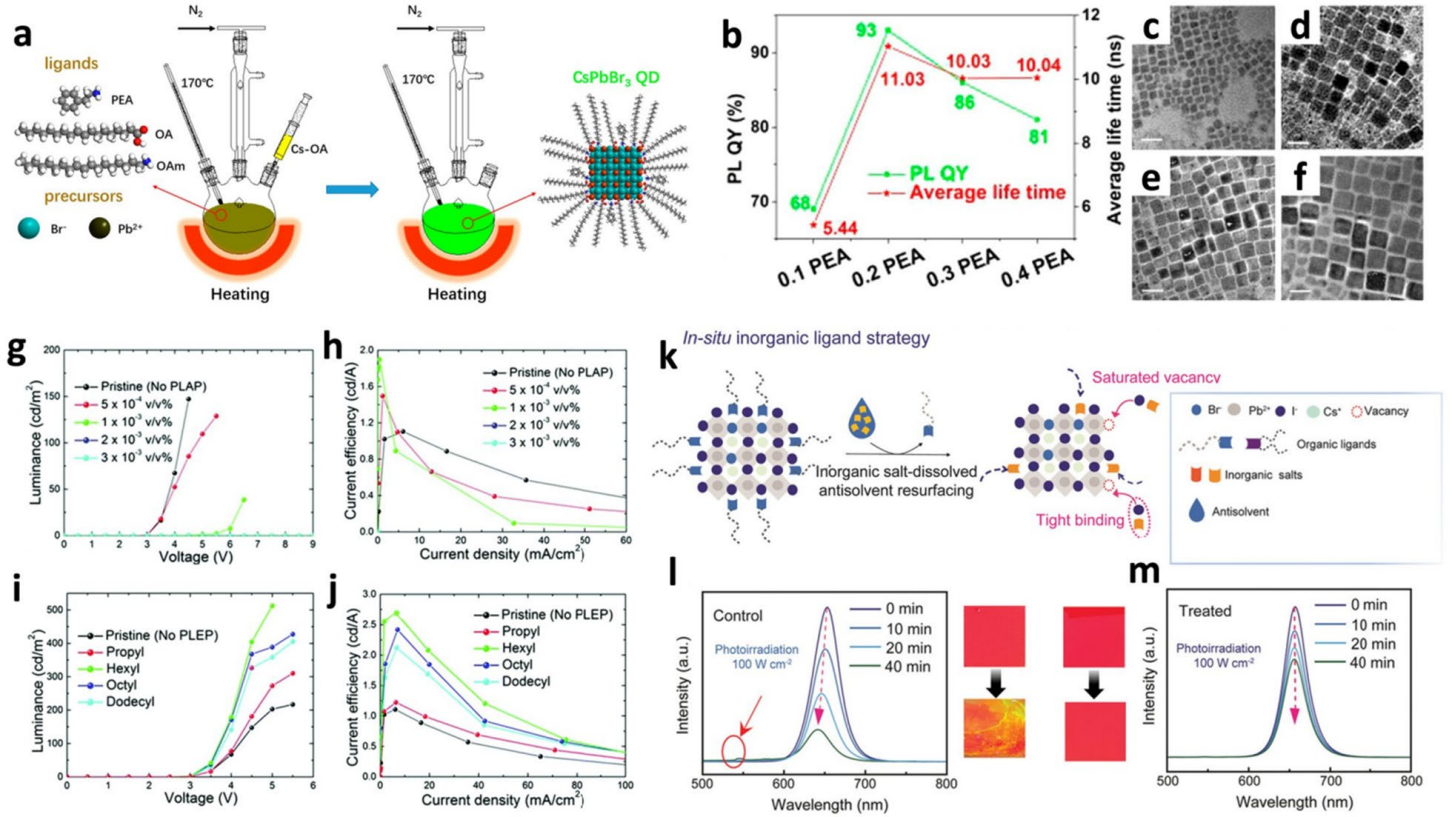
Ligand exchange (addition) processes, optical and device performances, microstructure and structural stabilities of PQDs. **a** Synthesis process of PEA modified CsPbBr_3_ QDs. **b** PLQY and average PL lifetime (τ_ave_) of CsPbBr_3_ QDs with different amounts of PEA. TEM images of PEA modified CsPbBr_3_ QDs; **(c)** 0.1, **(d)** 0.2, **(e)** 0.3, and **(f)** 0.4 molar fraction. Scale bar: 20 nm. Reproduced with permission from [51]. Copyright 2018, American Chemical Society. Luminance *vs*. voltage and current efficiency *vs*. current density curves of PeLEDs using CH_3_NH_3_PbBr_3_ QDs treated by the PLAP (**g** and **h**) and PLEP (**i** and** j**). Reproduced with permission from [53]. Copyright 2018, The Royal Society of Chemistry. **k** Process and advantage of the *in-situ* inorganic ligand strategy via a mildly polar anti-solvent. Bandgap stability of **(l)** control and (**m**) treated mixed-halide perovskite films under 100 Wcm^–2^ photo-irradiation. Photographs between **(l)** and **(m)** show the color change in degraded films after one week. Reproduced with permission from [45]. Copyright 2022, Wiley–VCH

As mentioned above, the surface defects of PQDs are caused by the steric hindrance of OA and OAm owing to their bent structures. In addition, the insulating properties of the long alkyl chain lengths of OA and OAm degenerate the device performance of PeLEDs, such as the external quantum efficiency (EQE) and luminance. Therefore, much efforts have been focused on improving the performance of PeLEDs by shortening the ligands of PQDs [50, 51]. Li et al*.* reported that the structural stabilities and electrical conductivities of CsPbX_3_ (X = Br, I) QDs were significantly improved via ligand exchange. The phenethylamine (PEA), π-conjugated ligand with a short chain length, was added into the Pb-pot for synthesizing CsPbX_3_ (X = Br, I) QDs using the hot-injection method [51]. PEA efficiently replaced OAm *in-situ* during the growth (fabrication) of the CsPbX_3_ (X = Br, I) QDs (Fig. 1a). Surface defects on the CsPbI_3_ QDs were effectively passivated with PEA. As the PEA content increased from a molar fraction of 0 to 0.2, the PLQY increased from 80 to 93%, and the average PL lifetime also increased from 7.28 ns to 11.03 ns (Fig. 1b). Conversely, the non-radiative recombination lifetime decreased from 0.028 to 0.006 ns. The EQE of the PeLED with 0.2 PEA CsPbI_3_ QDs reached 7.36%, which is almost three times higher than that of the PeLED with pristine CsPbI_3_ QDs (2.45%). This improvement in the device performance is attributed to the enhanced charge transport properties (electrical conductivities) of the CsPbI_3_ QD films through ligand exchange. Moreover, the additional post-treatment process of 0.2 PEA CsPbI_3_ QD film with a PEAI solution resulted in a higher concentration of PEA, not only improving the PLQY and structural stability, but also significantly improving the EQE of the PeLEDs by up to 14.08%. The π-conjugated benzene ring facilitated electronic coupling due to its shorter length, resulting in a more enhanced EQE as compared to OAm. However, in the case of CsPbBr_3_ QDs, when the PEA content increased from a molar fraction of 0 to 0.2, the radiative PL recombination lifetime of CsPbBr_3_ QDs decreased from 0.110 ns to 0.084 ns because quantum confinement weakened as the size of the PQDs increased [52]. As the PEA content increased from a molar fraction of 0.1 to 0.4, the size of CsPbBr_3_ QDs increased from 7.5 nm to 13 nm (Fig. 1c–f). When PQDs are fabricated using ligands with short alkyl chains, the crystal growth rate increases because of the high diffusion coefficient of the ligand, resulting in larger PQDs [41]. Therefore, post-treatment processes are considered more suitable than *in-situ* synthesis processes for the passivation of surface defects via ligand modification (exchange)*.*

Choi et al*.* reported the exchange of OA and OAm with ligands of different alkyl chain lengths to passivate surface defects through post-treatment process [53]. Effective surface passivation in CH_3_NH_3_PbBr_3_ QDs via a post-ligand addition process (PLAP) enhanced the PLQYs in both the solution and thin film states. PLAP is a post-treatment process in which small amounts of OA and OAm are added to passivate the surface defects caused by ligand dissociation on the surface of the PQDs during purification. When 1 × 10^–3^ v/v% of OA and OAm (volume ratio is 10:1) was added, PLQY increased from 80% (pristine CH_3_NH_3_PbBr_3_ in solution) to 95%. However, when a larger amount of ligand (3 × 10^–3^ v/v%) was added, the PLQY decreased to 72%. Efficient defect passivation at the optimal concentration (1 × 10^–3^ v/v%) resulted in a high PLQY and the structural stabilities of the CH_3_NH_3_PbBr_3_ QDs; however, the addition of excessive amounts of ligands induced aggregation of the PQDs and decreased the PLQY. As previously discussed, OA and OAm exhibit excellent surface passivation properties for PQDs; however, their insulating properties, owing to their long alkyl chain lengths, cause considerable limitations in PeLED applications that require high charge injection and transport properties. When ligands with short alkyl chains are used as passivation ligands, the electrical conductivities of PQDs can be significantly enhanced [52, 54, 55]. However, when ligands with short alkyl chain lengths were used to fabricate PQDs, nanosheets or nanorods were formed instead of zero-dimensional PQDs [40]. Therefore, PQDs were first synthesized using OA and OAm, followed by surface passivation via ligand exchange as a post-treatment process (*i.e.* post-ligand exchange process, PLEP). The PLQYs were improved by spin-casting short alkyl chain ligand solutions onto CH_3_NH_3_PbBr_3_ QD thin films [53]. The PLQYs of the ligand exchanged CH_3_NH_3_PbBr_3_ QD films (from 60 to 82%) were higher than that of pristine CH_3_NH_3_PbBr_3_ QD film (55%). The maximum PLQY was obtained when hexyl acid and hexylamine were used as the passivation ligands. When ligands with alkyl chain lengths shorter than hexyl were used for surface passivation, the alkyl chain length was too short to sufficiently passivate the surface defects. Conversely, ligands longer than hexyl were unable to penetrate the PQDs thin film, resulting in less surface passivation. The device performance of PeLEDs is affected by the PLQYs and electrical conductivities of PQDs, which depend on the type of ligands used for surface passivation. As the amount of added ligands (OA and OAm) increased in the PLAP process, the defects on the surface of the CH_3_NH_3_PbBr_3_ QDs were healed, resulting in improved PLQYs. However, the insulating properties of OA and OAm deteriorated the charge transport properties of PQDs, leading to poor device performance in terms of current efficiency and luminance (Fig. 1g, h). On the other hand, the average inter-particle distance decreased when the surface ligands of the CH_3_NH_3_PbBr_3_ QDs were exchanged with shorter alkyl chain ligands via PLEP. This increased the packing density of the CH_3_NH_3_PbBr_3_ QDs in the film, resulting in improved charge carrier mobility and device performance (Fig. 1i, j).

In addition to the organic ligands, Wang et al*.* reported ligand exchange (modification) using inorganic ligands [45]. Ethyl acetate is commonly used as an anti-solvent for the purification of CsPb(I_x_Br_1-x_)_3_ QDs. During the purification, *in-situ* ligand exchange with inorganic ligands was conducted by dissolving potassium iodide in ethyl acetate (Fig. 1k**)**. The red emissive mixed-halide perovskite, CsPb(I_x_Br_1-x_)_3_ QDs, easily decompose under ambient conditions, resulting in a decrease in the PLQY and a band gap shift (PL emission shift). The structural degradation of the PQDs is caused by an imbalance in the halide composition, which is attributed to the energy difference between Br and I for ion migration [56]. The short inorganic ligands are in the form of halogen ions, which effectively passivate surface defects caused by halide vacancies in PQDs. In addition, PQDs passivated by inorganic ligands are much more structurally stable than those passivated by organic ligands because the halogen (iodine) and metal (potassium) ions in the inorganic ligands form tight bonds. As a result, ligand exchange with inorganic ligands can stabilize both the crystal structure and bandgap of PQDs by suppressing the formation of halide vacancies due to exposure to external stimuli, such as light irradiation or heat (Fig. 1l, m).

### Core–shell structure

Recently, there has been extensive research on core–shell structures, which are widely utilized in II-IV semiconducting nanoparticles (CdSe/ZnS, CdTe/CdS, CdTe/CdSe), to improve the structural stabilities of PQDs against external stimuli. Materials for shells are categorized as organic (*e.g.,* polymers) and inorganic materials (*e.g.,* oxide materials). Organic materials used for PQD shells have a high resistance to the environment, especially moisture; however, they have low resistance to heat [57, 58]. On the other hand, inorganic materials are more thermally stable than organic materials, and various oxide materials, including TiO_2_ and SiO_2_, can be used to form shells [59]. Among various inorganic oxide materials, SiO_2_ is the most commonly used. However, the fabrication of core–shell (SiO_2_) PQDs via post-treatment process has drawbacks that must be addressed, such as the aggregation of PQDs and the presence of multiple PQDs within a single SiO_2_ shell, making them unsuitable for LED applications [60]. Therefore, developing novel methods in which each PQD is coated with a SiO_2_ shell without aggregation is necessary.

Gao et al*.* fabricated core–shell structured SiO_2_ coated CsPbBr_3_ QDs by post-treatment process of CsPbBr_3_ QDs with tetraethylorthosilicate (TEOS) as a SiO_2_ precursor [61]. TEOS with non-polar functional groups was chosen as the SiO_2_ precursor to minimize the structural degradation of the PQDs during post-treatment process. The SiO_2_ shell was formed via the hydrolysis of TEOS under ambient conditions without additional polar solvents to control the rate of the hydrolysis reaction (the hydrolysis reaction only occurs when the silane precursor is exposed to moisture or polar solvents). However, when the SiO_2_ shell was formed using TEOS, the hydrolysis reaction rate was uncontrolled and processed rapidly, resulting in the aggregation of the SiO_2_ matrix. In this study, monodisperse core–shell CsPbBr_3_@SiO_2_ QDs (*i.e.,* a PQD is coated with a SiO_2_ shell) were fabricated by adding trioctylphosphine oxide (TOPO) with TEOS to prevent multiple PQDs from being encapsulated by the SiO_2_ shell. TOPO acts as a ligand to passivate the surface of the CsPbBr_3_ QDs and to prevent the PQDs from being directly exposed to moisture. This effectively reduced the hydrolysis rate of TEOS, enabling the fabrication of aggregation free core–shell CsPbBr_3_@SiO_2_ QDs in which a PQD is coated by a single SiO_2_. The PLQY of the core–shell CsPbBr_3_@SiO_2_ QDs was 87%, which is higher than that of core–only CsPbBr_3_ QDs (75%), owing to the effective surface passivation by the SiO_2_ shell.

**Fig. 2:**
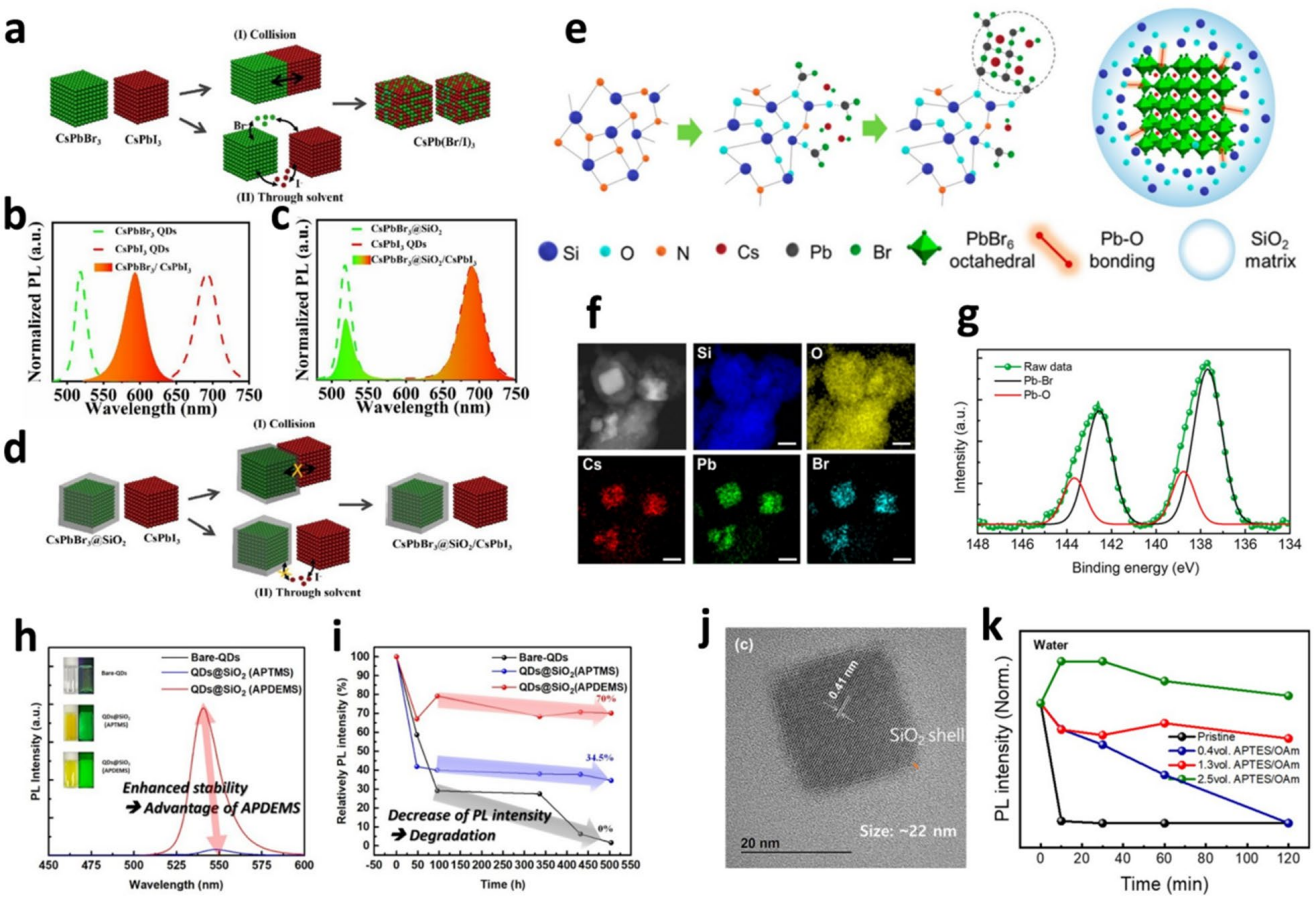
Anion exchange processes between PQDs, *in-situ* fabrication of core–shell PQDs and characterization, structural stabilities of core–shell PQDs under external stimuli. **a** Schematic of anion exchange process between core–only PQDs with different halide ions. **b** PL spectra of core–only CsPbBr_3_ QDs solution, core–only CsPbI_3_ QDs solution, and their mixed solution after mixing for 2 h. **c** PL spectra of core–shell CsPbBr_3_@SiO_2_ QDs solution, core–only CsPbI_3_ QDs solution, and their mixed solution after mixing for 2 h. **d** Schematic of preventing the anion exchange process between core–shell CsPbBr_3_@SiO_2_ QDs and core–only CsPbI_3_ QDs. Reproduced with permission from [61]. Copyright 2021, Elsevier. **e** Schematic of *in-situ* fabrication of SiO_2_ encapsulated CsPbBr_3_–PSZ composites. **f** TEM image and EDS elemental mapping of CsPbBr_3_–PSZ composites. Scale bar: 100 nm. **g** XPS spectra of CsPbBr_3_–PSZ composites. Reproduced with permission from [62]. Copyright 2023, American Chemical Society. **h** Comparison of the dispersion stability from the PL spectra and real images of bare-QDs and QDs@SiO_2_ core/shell samples in a polar solvent (acetone) environment. Thermal stability: **i** PL intensity as a function of the time of heat exposure (at 60 °C). Reproduced with permission from [63]. Copyright 2021, Elsevier. **j** TEM images of A-CsPbBr_3_@SiO_2_ QDs with APTES/OAm volume ratio of 1.3. **k** Chemical resistance of pristine CsPbBr_3_ QDs and A-CsPbBr_3_/@SiO_2_ QDs with APTES/OAm volume ratios of 0.4, 1.3, and 2.5 in water. Reproduced with permission from [65]. Copyright 2021, American Chemical Society

Simple halide exchange via the anion exchange process causes color purity issues in white displays through the stacking or mixing of red, green, and blue PQDs (Fig. 2a, b). As shown in Fig. 2b, when core–only CsPbBr_3_ and core–only CsPbI_3_ QDs solutions were mixed, the respective PL spectra of the PQDs disappeared, and a new PL spectrum appeared, which was located between the respective PL spectra of the core–only CsPbBr_3_ and core–only CsPbI_3_ QDs. However, when the PQDs were coated with a SiO_2_ shell, anion exchange was effectively prevented (Fig. 2c, d). When the core–shell CsPbBr_3_@SiO_2_ and the core–only CsPbI_3_ QDs solutions were mixed, their respective PL spectra were well maintained (Fig. 2c). These results demonstrate that the application of core–shell PQDs in various devices (*e.g.,* LEDs) can suppress not only the deterioration of the optoelectronic properties of PQDs, but also their structural degradation caused by the anion exchange process. In addition, to investigate the effect of the SiO_2_ shell on the structural stabilities of PQDs subject to heat and moisture, the PL intensity changes of core–shell CsPbBr_3_@SiO_2_ QDs were measured when exposed to 120 °C and atmosphere for up to 30 days, respectively. The PL intensity of core–only CsPbBr_3_ QDs was completely quenched at 120 °C and after 25 days under ambient conditions, whereas the PL intensity of core–shell CsPbBr_3_@SiO_2_ QDs remained at 85% of its initial PL intensity at 120 °C and 90% of its initial value after 30 days under ambient conditions. Additionally, approximately 80% of its initial PL intensity was retained for up to 168 h under UV irradiation (365 nm) and up to 8 days in pure water.

Park et al*.* reported SiO_2_ encapsulated CsPbBr_3_–PSZ composites via* in-situ* synthesis using perhydropolysilazane (PSZ) without organic ligands (OAm and OA) [62]. When a mixed solution of CsBr, PbBr_2_, and PSZ is dropped onto a substrate at 100 °C, CsPbBr_3_-PSZ composites are fabricated through the evaporation of the solvent. As the solvent evaporates, the PSZ (silicon nitride (SiN_x_)) reacts with moisture in the air to form SiO_2_. Simultaneously, the Pb in CsPbBr_3_ and O in SiO_2_ form stable covalent bonds, resulting in the synthesis of a green CsPbBr_3_–PSZ composite powder, which takes 60 s, longer than that of the conventional LARP and hot-injection methods. This process allowed the fabrication of SiO_2_ encapsulated CsPbBr_3_–PSZ composites without organic ligands (Fig. 2e). Transmission electron microscopy-energy dispersive spectroscopy (TEM-EDS) mapping analysis revealed that the CsPbBr_3_–PSZ composites were encapsulated by SiO_2_, which was confirmed by the presence of Si and O in the composites (Fig. 2f). In particular, the Pb–O and the Pb–Br binding peaks in the X-ray photoelectron spectroscopy (XPS) analysis demonstrated that the SiO_2_ shell did not physically surround the CsPbBr_3_, but was chemically bonded to the surface of CsPbBr_3_ to form the CsPbBr_3_–PSZ composites (Fig. 2g). The CsPbBr_3_–PSZ composite retained more than 60% of its initial PL intensity even after long-term storage in water for more than 1100 h, which is more than 600 times longer than that of core–only CsPbBr_3_ QDs fabricated by the conventional hot-injection method.

Recently, many studies have reported the *in-situ* fabrication of core–shell PQDs. The fabrication of core–shell PQDs via an *in-situ* process is simple, and it alleviates the problems that arise during SiO_2_ shell formation by post-treatment process. Kim et al. synthesized core–shell CH_3_NH_3_PbX_3_@SiO_2_ (X = Cl, Br and I) QDs *in-situ* using the LARP method with the addition of 3-aminopropyltrimethoxysilane (APTMS) and 3-aminopropyldiethoxymethylsilane (APDEMS) [63]. APTMS and APDEMS, which have amine functional groups, differ only in the number of methoxy groups (–OCH_3_) and have been used as precursors for SiO_2_ shell formation. APTMS has three methoxy groups, whereas APDEMS has two methoxy groups and one methyl group (–CH_3_). CH_3_NH_3_PbX_3_@SiO_2_ (X = Cl, Br and I) QDs can be synthesized using APTMS or APDEMS, where the two silane groups act as surface passivation ligands instead of OAm and form a SiO_2_ shell via the hydrolysis reaction. When APTMS with three Si–O bonds was used as the silane precursor, the core–shell PQDs easily aggregated because of the presence of –SiOH groups that remained after the hydrolysis reaction. Meanwhile, when APDEMS with two Si–O bonds was used as the silane precursor, the core–shell PQDs did not aggregate because of the absence of unreacted –SiOH groups. After the hydrolysis reaction, the SiO_2_ shell was terminated by the –CH_3_ groups rather than the –OH group, and the Si–CH_3_ groups suppressed aggregation by allowing the core–shell PQDs to disperse well in non-polar solvents. These results clearly demonstrate that the number of silane groups in the precursor is critical for achieving monodispersed core–shell PQDs without aggregation by controlling the dispersibility. To verify the structural (dispersion) stabilities of the core–shell PQDs subject to polar solvents and heat, the PL intensity changes of core–shell CH_3_NH_3_PbBr_3_@SiO_2_ QDs were measured after they are dispersed in acetone (Fig. 2h) and exposed to 60 °C for 500 h, respectively (Fig. 2i). In polar solvents, core–only PQDs (bare-QDs) exhibited low PL intensity, whereas core–shell PQDs (QDs@SiO_2_ (APDEMS)) maintained high PL intensity. Furthermore, the bare-QDs showed no PL emission after 500 h under 60 °C, whereas QDs@SiO_2_ (APTMS) and QDs@SiO_2_ (APDEMS) maintained 34.5% and 70% of their initial PL intensity, respectively.

The structural stabilities of core–shell PQDs can be further improved by increasing the SiO_2_ shell thickness. However, the electrical properties (*i.e.,* charge injection and transport) of core–shell PQDs decrease with increasing SiO_2_ shell thickness owing to the insulating properties of SiO_2_ [64]. Therefore, fabricating core–shell PQDs coated with an ultra-thin SiO_2_ shell (< ~ 2–3 nm) is necessary to ensure effective charge injection and transporting for PeLED applications. Recently, Trinh et al*.* reported core–shell CsPbBr_3_@SiO_2_ QDs via an *in-situ* hot-injection process using 3-aminopropyl-triethoxysilane (APTES) as an amine ligand and precursor for SiO_2_ shell formation [65]. Generally, nanocrystals are grown thermodynamically and kinetically. Under kinetic growth conditions, the nanocrystals grow into large uniform particles via Ostwald ripening and/or gradual crystal growth. Meanwhile, when nanocrystals grow thermodynamically, crystal reconstruction occurs during the crystal growth process, resulting in energetically stable products. The amine group of APTES strongly binds to the surfaces of the PQDs and slows down the surface ligand dissociation rate of OA, which reduces the adsorption rate of metal–Br to the surfaces of the PQDs and the growth rate of the PQDs. For this reason, it takes about 20 s to fabricate core–shell CsPbBr_3_@SiO_2_ QDs, which is a long time compared to that of pristine CsPbBr_3_ QDs (~ 5 s). In this work, core–shell CsPbBr_3_ QDs coated with an ultra-thin SiO_2_ shell (~ 2 nm) were successfully fabricated via an *in*-*situ* process rather than post-treatment process (Fig. 2j). As the amount of APTES, which acts as an amine ligand and a SiO_2_ precursor, increased, the size of the core–shell CsPbBr_3_@SiO_2_ QDs increased from 11 to 27 nm, and their PLQYs increased from 45 ± 5% to 70 ± 5%. The trap density of core–shell CsPbBr_3_@SiO_2_ QDs decreased as a result of the surface passivation by the SiO_2_ shell and A–site doping with APTES, which led to an increase in the PLQY and an average PL lifetime compared to pristine CsPbBr_3_ QDs. The core–shell CsPbBr_3_@SiO_2_ QDs exhibited a charge mobility of ~ 1.39 × 10^–3^ cm^2^/Vs (for a 1.3 volume ratio of APTES/OAm), which is similar to that of core–only CsPbBr_3_ QDs (~ 1.92 × 10^–3^ cm^2^/Vs). As shown in Fig. 2k, the core–shell CsPbBr_3_@SiO_2_ QDs exhibited high structural stabilities to polar solvents, such as isopropyl alcohol, ethanol, and water, especially with increasing thickness of the SiO_2_ shell. These results demonstrate that the ultra-thin SiO_2_ shell contributes significantly to improving the structural stabilities of PQDs but does not affect the charge balance for PeLED applications. The PeLEDs fabricated with core–shell CsPbBr_3_@SiO_2_ QDs (for a 1.3 volume ratio of APTES/OAm) exhibited a maximum luminance of ~ 3,200 cd/m^2^ at 830 mA/cm^2^. Furthermore, the half-life of the core–shell CsPbBr_3_@SiO_2_ QDs based PeLEDs (initial condition; 100 cd/m^2^) under ambient conditions was 43 min, which is significantly better than that of PeLEDs with pristine CsPbBr_3_ QDs. The high structural stabilities of the core–shell CsPbBr_3_@SiO_2_ QDs in polar solvents enabled the fabrication of PeLEDs through all-solution processes.

### Crosslinking

The use of crosslinkable ligands can significantly improve the structural stabilities of PQDs by suppressing ligand dissociation on their surfaces. Furthermore, altering the solubility of the PQDs enables the patterning and stacking of films, which can be applied to various optoelectronic devices. This process consists of (1) replacing OA and OAm ligands with long alkyl chains on the surface of PQDs with crosslinkable ligands through ligand exchange processes, and (2) inducing the crosslinking of the ligands through post-treatment processes such as UV and thermal irradiation (heat).

**Fig. 3:**
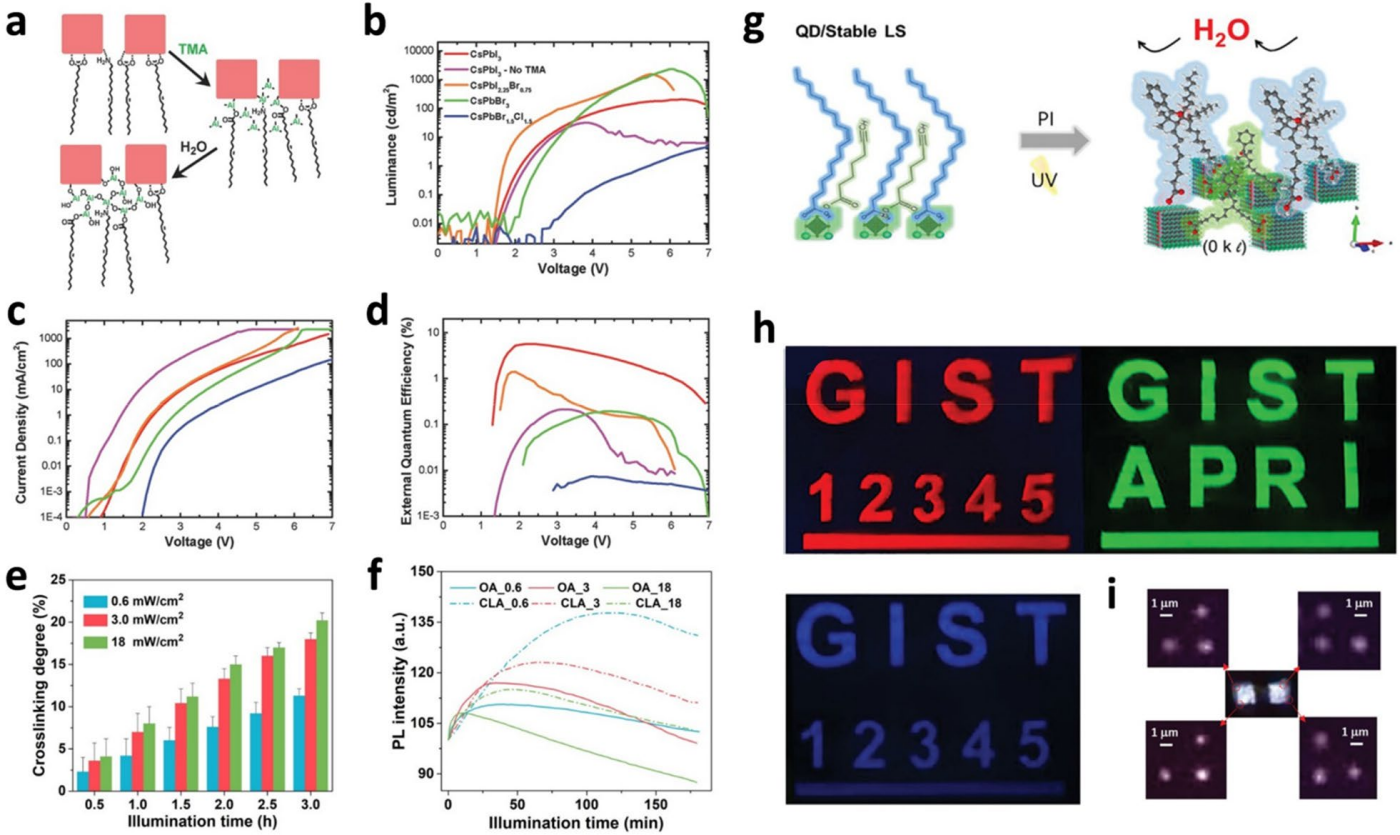
Crosslinking processes, device performances and PQDs patterning. **a** Reaction schematic of TMA crosslinking process. **b** Luminance *vs*. voltage, **(c)** current density *vs*. voltage, and **(d)** EQE *vs*. voltage curves of PeLEDs using crosslinked PQDs as an emissive layer. Reproduced with permission from [66]. Copyright 2016, Wiley–VCH.** e** Crosslinking degree of CLA-capped CsPbBr_3_ QDs films as a function of UV irradiation intensity and exposure time.** f** PL intensity of CsPbBr_3_ QDs and of CLA-capped CsPbBr_3_ QDs films upon continuous illumination time. Reproduced with permission from [68]. Copyright 2020, American Chemical Society.** g** Schematic of degradation mechanism of UV crosslinked QD/Stable LS film.** h** Fluorescence images of ink-jet printed patterns with red, green, and blue QD/Stable LS. **i** EL images of ~ 1 μm patterned white emissive PeLEDs with green and red QD/Stable LS. Reproduced with permission from [71]. Copyright 2021, Wiley–VCH

The introduction of crosslinkable ligands into CsPbX_3_ (X = Cl, Br and I) QDs was first reported by the Greenham and Tan groups [66]. In this study, the crosslinking of PQDs was realized by exposing PQD films to trimethylaluminum (TMA) vapor via atomic layer deposition (ALD) at room temperature. In general, exchanging long alkyl chain ligands with crosslinkable ligands through PLEP could cause cracks or holes in the film, and PL self-quenching might be induced when PQDs are packed at a high density [67]. Therefore, it is desirable to maintain the initial states (*e.g.,* crystal structure, morphology, etc*.*) of the PQDs by retaining the existing ligands with long alkyl chain lengths (*e.g.,* OAm and OA used in PQDs fabrication) when PQDs are crosslinked in the film. The crosslinking of PQDs using TMA was achieved without an additional ligand exchange process using hydroxide-terminated aluminum oxide (AlO_x_) to form a matrix between the ligands of the PQDs. In detail, the TMA-coated PQDs obtained through the ALD process are exposed to moisture in the air and hydrolyzed to alumina and/or aluminum hydroxide, which covalently bonds with the ligands of PQDs, resulting in crosslinking (Fig. 3a). In addition to crosslinking, aluminum oxide also passivates the surface defects in the PQDs, contributing to improved structural stabilities. Consequently, high-quality PeLEDs can be realized by stacking a hole injection layer (TFB) via a solution process without damaging the PQDs emissive layer (Fig. 3b–d). Compared to that of untreated CsPbI_3_ QDs, the current density of PeLEDs with TMA-treated CsPbI_3_ QDs was more than 10 times lower despite having similar luminance, indicating that the TMA-treated CsPbI_3_ QD layer had relatively fewer voids. As a result, the EQE of the PeLED with the TMA-treated CsPbI_3_ QDs was 5.7%, which was considerably higher than that of the PeLED with the untreated CsPbI_3_ QDs.

Wei et al*.* reported crosslinked CsPbBr_3_ QDs films using conjugated linoleic acid (CLA) as a photo-crosslinking ligand by optimizing the UV irradiation intensity and exposure time. Furthermore, the structural stabilities of the PQDs were investigated by monitoring the change in PL intensity with water exposure time [68]. CLA, a derivative of polyunsaturated omega-6 fatty acids, contains carboxyl functional groups, which can efficiently passivate surface defects in PQDs as well as provide colloidal stabilities to PQDs, such as OA. The conjugated olefin (alkene) bonds of CLA can be crosslinked via photopolymerization. [69]. In addition, the low price of CLA enables its wide commercially use as a photo-crosslinking ligand [70]. The degree of CLA crosslinking as a function of UV irradiation intensity and exposure time was investigated (Fig. 3e). The degree of crosslinking increased proportionally with increasing UV irradiation intensity and exposure time. More specifically, there was a large difference (11.3% to 18% at 3 h) in the degree of crosslinking when the UV irradiation intensity was 0.6 mW/cm^2^ and 3 mW/cm^2^, but little difference (18% to 20% at 3 h) between 3 mW/cm^2^ and 18 mW/cm^2^. This suggests that the degree of crosslinking saturates above a particular UV irradiation intensity. The structural (photo) stabilities of the CsPbBr_3_ QDs passivated by different types of ligands (*i.e.*, OA and CLA) were investigated through changes in the PL intensities with UV exposure time (Fig. 3f). After UV irradiation, CsPbBr_3_ QDs passivated with OA were aggregated owing to ligand desorption, resulting in a decrease in PL intensity. Compared to the CsPbBr_3_ QDs passivated by OA, the CsPbBr_3_ QDs passivated by CLA exhibited high structural stabilities after UV irradiation and maintained a significantly high PL intensity for a long time without degradation. The crosslinked CsPbBr_3_ QDs film retained 67% of its initial PL intensity after immersion in water for 3 h and up to 80% when stored for 30 days under ambient conditions. To verify that the transport layer could be deposited on the crosslinked PQD films via the solution process, washing tests were conducted with a non-polar solvent such as chlorobenzene (CB). After rinsing with CB, no dissolution or damage to the PQDs was observed, and the morphology of the film remained in its initial state. These results clearly demonstrate that the crosslinking of PQDs enables the fabrication of highly efficient and stable PeLEDs by stacking an electron transport layer via a solution process.

Lee et al. reported highly stable CsPbX_3_ (X = Cl, Br, and I) QDs obtained through photo-crosslinking under UV light using 2-hydroxy-2-methylpropiophenone as a photoinitiator and alkynoic acids with alkyne (C≡C) groups as crosslinking ligands. Crosslinked PQDs enable micro-scale patterning through changes in solubility in solvents and the realization of PeLEDs through ink-jet printing [71]. When the photoinitiator is exposed to UV light, radical carbonyl groups are generated, and radicals on the surface ligands of the PQDs can propagate through bond breaking. Thus, in this study, the alkyne (C≡C) groups of alkynoic acid were crosslinked to form a chemically stable matrix (Fig. 3g). The PL intensity of the CsPbBr_3_ QD (QD/stable ligand system (LS)) film was significantly enhanced by crosslinking via UV irradiation. Alkynoic acids with short alkyl chain lengths diffused more efficiently because of less steric hindrance, thereby enabling them to bind more effectively to the surface of the CsPbBr_3_ QDs compared to bulky OA. Moreover, physically adsorbed alkynoic acids were converted to chemically bound surface ligands via photo-crosslinking, effectively healing surface defects. The enhanced structural stabilities of the CsPbBr_3_ QDs (QDs/stable LS) were confirmed by the absence of the Pb^0^ peak in the XPS analysis and longer radiative recombination lifetime in PL decay curves. The green emissive PeLED exhibited a high luminance of 9,700 cd/m^2^ at 370 mA/cm^2^ and a current efficiency of ~ 6.7 cd/A at 100 cd/m^2^. High-resolution patterning was achieved using simple photolithography without a photoresist or ink-jet printing. Materials must have high chemical and structural stabilities to be used in ink-jet printing processes. The PQDs (QDs/stable LS) developed in this study perfectly fulfilled these requirements. As shown in Fig. 3h, ink-jet printing with CH_3_NH_3_PbI_3_ QDs (red), CH_3_NH_3_PbBr_3_ QDs (green), and CH_3_NH_3_PbBr_x_Cl_3-x_ QDs (blue) produced letters, numbers, and lines with high brightness. Finally, high-resolution (~ 1 μm) white PeLEDs were successfully fabricated via the ink-jet printing of green and red emitting PQDs with high chemical stabilities (Fig. 3i). The realization of high-resolution PeLEDs through all-solution processes enables the low-cost production of next-generation displays.

### Metal doping

In addition to surface defect passivation by ligand modification, many recent studies have reported that metal ion doping (*e.g*., alkali metal doping) can improve the structural stabilities of PQDs by suppressing the formation of intrinsic defects (*i.e.*, defects in perovskite core) which are generated during PQDs fabrication. Metal ion doping reduces the defect density in PQDs, which also enhances their optoelectronic properties, such as PLQYs [72]. The crystal structure of the perovskite is determined by the Goldschmidt tolerance factor *t*, which is described as follows: [73]

$$t= \frac{{r}_{A}+{r}_{X}}{\sqrt{2}\left({r}_{B}+{r}_{X}\right)}$$

where *r*_*A*_, *r*_*B*_, and *r*_*X*_ are the radius of the A–, B–, and X–site ions, respectively. The perovskite crystal structure only forms when the tolerance factor is within the range of 0.76–1.13 [74]. Furthermore, the octahedron factor (*μ* = *r*_*B*_/*r*_*X*_), which determines the structural stability of the [BX_6_]^4−^ octahedron, must be within the range of 0.442–0.895 [73]. Metal ion doping can be categorized into three groups based on (1) the number of ionic charge (*e.g.,* monovalent, divalent and trivalent), (2) elemental type (*e.g.,* alkali metal, lanthanide and alkaline earth metal, etc*.*), and (3) doping site (*e.g.,* A–, B–, and X–sites) [75–86]. Notably, when metal ions are doped into PQDs, both the tolerance and octahedral factors need to be considered. Many studies have reported that the A– and B–sites of PQDs can be doped with various metal ions (cations) of different charge numbers and elements [77, 80–86]. When metal ions with ionic radius similar to those of the doping sites (*i.e*., A– or B–site ions) are used as dopants, the structural stabilities of PQDs can be significantly improved by changing the B–X bond length [87, 88].

**Fig. 4:**
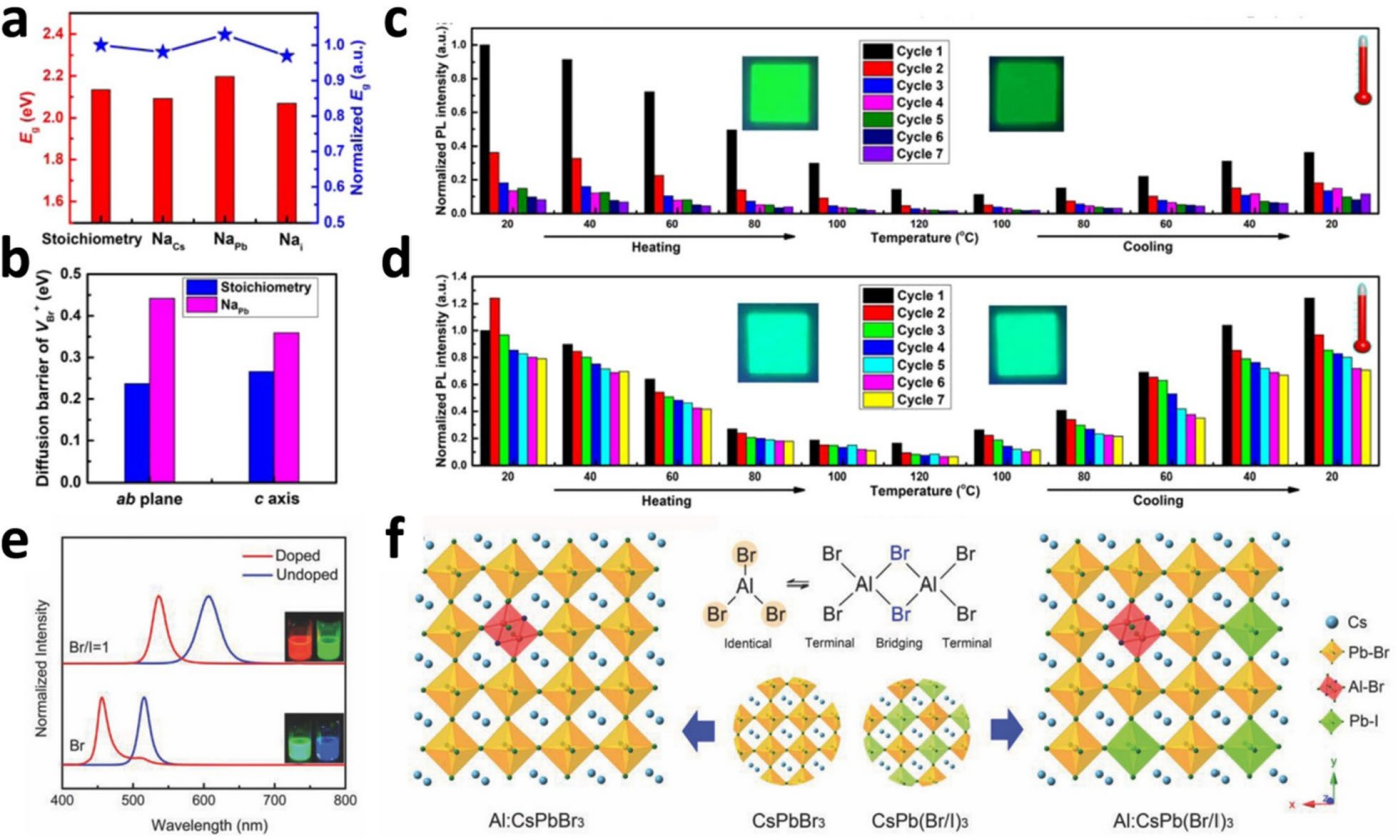
DFT calculations, thermal stabilities and bandgap engineering of metal doped PQDs. **a** Calculated and normalized bandgap (E_g_) of CsPbBr_3_ QDs with and without defects. **b** Calculated diffusion barrier energy of V_Br_ as diffusion paths. Thermal stabilities of **(c)** CsPbBr_3_ QDs and **(d)** Na^+^ doped CsPbBr_3_ QDs in seven thermal cycling measurements. The insets show photographs of the two samples under UV irradiation before and after the seven thermal cycling measurements. Reproduced with permission from [80] Copyright 2019, American Chemical Society. **e** PL spectra of Al^3+^ doped and undoped CsPbBr_3_ and CsPb(Br/I)_3_ QDs. Insets are photographs of the sample under UV excitation. **f** Schematics showing the Al bound to CsPbBr_3_ or CsPb(Br/I)_3_ lattice constituents in cluster form. Reproduced with permission from [90]. Copyright 2017, Wiley–VCH

Li et al*.* reported that *in-situ* Na^+^ doping in LARP improved the PLQYs and color purities of CsPbBr_3_ QDs as well as the blue-shift of PL emission [80]. Density functional theory (DFT) calculations showed that Na^+^ energetically favored Na_Pb_ substitution, thereby increasing the bandgap and diffusion barrier of the Br vacancies. The X-ray diffraction (XRD) results showed that the peaks shifted to higher angles (*i,e*., a decrease in lattice parameter) as the Na^+^ doping concentration increased. This is consistent with the DFT results, suggesting that Na ions were doped into the lattice of the CsPbBr_3_ QDs, particularly at the Pb^2+^ sites (Pb^2+^ substitution). Furthermore, TEM–EDS elemental mapping clearly showed that Na ions were uniformly distributed throughout the CsPbBr_3_ QDs. The PLQYs of the Na^+^ doped CsPbBr_3_ QDs increased with increasing Na^+^ doping concentration. In particular, the PLQY of Na^+^ doped CsPbBr_3_ QDs with a Na/Pb ratio of 1.25 was 85%, which was approximately twice that of pristine CsPbBr_3_ QDs. Moreover, the crystallinity and particle size distribution were significantly improved in the Na^+^ doped CsPbBr_3_ QDs. Notably, the PL spectrum of the Na^+^ doped CsPbBr_3_ QDs is blue-shifted with increasing Na^+^ doping concentration. The bandgap calculations for Na_Cs_, Na_Pb_, and Na_i_ defects in Na^+^ doped CsPbBr_3_ QDs revealed that only the bandgap of Na_Pb_ increased relative to the stoichiometry (defect-free, undoped) (Fig. 4a). The calculation results are consistent with the PL analysis results, which was confirmed that Na^+^ substituted the Pb^2+^ sites, increasing the bandgap and shifting the PL peak to blue. The Na^+^ doped CsPbBr_3_ QDs maintained 75% of their initial PL intensity after 35 h of exposure to UV light (365 nm) and 85% after 20 min of exposure to water. This is because the Na_Pb_ formed in CsPbBr_3_ QDs through Na^+^ doping not only increases the diffusion barrier energy of V_Br_, but also improves the structural stabilities of the PQDs by slowing structural degradation (Fig. 4b). The thermal stabilities of Na^+^ doped CsPbBr_3_ QDs were investigated using thermal cycling measurements (Fig. 4c, d). After being heated to 120 °C and then cooled to room temperature, pristine CsPbBr_3_ QDs showed a 60% decrease in PLQY, whereas CsPbBr_3_ QDs with a Na/Pb ratio of 1.25 showed a 25% increase in PLQY. After seven cycles, pristine CsPbBr_3_ QDs exhibited a 90% decrease in PLQY, whereas CsPbBr_3_ QDs with a Na/Pb ratio of 1.25 showed only a 30% decrease in PLQY, indicating that the thermal stabilities of the PQDs were significantly improved by Na^+^ doping.

Recently, many studies have focused on doping potassium ions (K^+^) into CsPbBr_3_ QDs [81]. The gradual shift of the XRD pattern to a lower angle with increasing K^+^ doping concentration and DFT calculation results suggested that doped K^+^ occupies the interstitial sites of the CsPbBr_3_ QDs (*i.e.,* they becomes an interstitial defect) [81, 89]. The PLQYs of the K^+^ doped CsPbBr_3_ QDs increased with increasing K^+^ doping concentration owing to the enhanced radiative exciton recombination. K^+^ doping is also very effective in enhancing the structural stabilities of PQDs. When stored under ambient conditions, the PL intensity of pristine CsPbBr_3_ QDs decreased rapidly, whereas that of K^+^ doped CsPbBr_3_ QDs barely decreased even after 7 days. The microstructures of the CsPbBr_3_ QDs were investigated after 9 days of exposure to air. Considerable aggregation occurred in the pristine CsPbBr_3_ QDs, whereas no aggregation was observed in the K^+^ doped CsPbBr_3_ QDs.

The charge of the dopants should be the same as that of the ions in the A– and/or B– sites of the PQDs. Liu *et. al.,* reported the effective bandgap engineering of PQDs by substituting Pb^2+^ (divalent) at the B–site with Al^3+^ (trivalent) with different charge number [90]. Generally, the valence band of a Pb based PQD is determined by the *p*-orbitals of the halide, whereas the conduction band is formed by the *p*-orbitals of Pb [91]. Therefore, when Al^3+^ is doped into CsPbBr_3_ QDs, a new energy band was formed through the hybridization of the *s*-orbital of Al, *p*-orbital of Br, and *p*-orbital of Pb, resulting in a large shift in the PL spectra (from 515 to 456 nm and from 607 to 536 nm for CsPb(Br/I)_3_) (Fig. 4e). Moreover, Pb^2+^ (B site) was substituted by Al^3+^, and the [PbBr_6_]^4−^ octahedron was replaced with the dimeric form of aluminum tribromide (Al_2_Br_6_) (Fig. 4f). Because the ionic radius of Al^3+^ (53.5 pm) is considerably smaller than that of Pb^2+^ (119 pm), the lattice distortion is not severe; thus, the perovskite structure is well maintained. Nevertheless, slight distortion and tilting of the [PbBr_6_]^4−^ octahedron were observed in the XRD pattern and XPS binding energy analysis, which confirmed that the Al:CsPbBr_3_ QDs were slightly contracted compared to CsPbBr_3_ QDs. This result showed that the length of the Pb–Br bond was reduced by Al^3+^ doping, suggesting a stronger interaction between Pb^2+^ and the halide. To investigate the thermal stabilities of Al:CsPbBr_3_ and CsPbBr_3_ QDs films, changes in PL intensity under the cyclic processes of raising the temperature to 100 °C and then cooling to room temperature were investigated. For the CsPbBr_3_ QDs, the PL intensity decreased to 10% of its initial value at 100 °C, but it recovered to 60% upon returning to room temperature. On the other hand, for Al:CsPbBr_3_ QDs, the PL intensity decreased to 40% of its initial value at 100 °C, but it recovered to almost its initial value upon returning to room temperature.

## Conclusions

This review presents various methods to improve the structural stabilities of PQDs. The strategies introduced herein improve the poor structural stabilities of PQDs owing to their intrinsic ionic properties, thereby enabling their commercial use in PeLEDs. The four representative strategies are (1) ligand modification, (2) core–shell structure, (3) crosslinking, and (4) metal doping. The experimental methods, objectives, advantages, and disadvantages of each strategy as well as the enhanced structural stability of the PQDs are discussed.

(1) Ligand modification involves the exchange of conventional ligands with long alkyl chains (*e.g.,* OA and OAm) with relatively shorter or conjugated ligands. When long alkyl chain ligands are exchanged with shorter or conjugated ligands, not only the charge carrier mobility is enhanced, but the surface defect density is also reduced owing to the enhanced ligand packing. As a result, the structural degradation of the PQDs due to exposure to external stimuli can be suppressed. (2) The core–shell structure involves the introduction of polymers or inorganic materials (*e.g.,* oxide materials) as shell layers to improve the structural stabilities of the PQDs against external stimuli (moisture or oxygen). Core–shell perovskite@SiO_2_ QDs have been fabricated via the post-treatment of silane derivatives (*e.g.*, TEOS and APDEMS) or *in-situ* processes (APTES) during PQDs fabrication. The structural stabilities of PQDs coated with ultra-thin SiO_2_ shells (< ~ 2–3 nm) have been dramatically improved without compromising their electrical properties (*i.e.,* charge injection and transport) for PeLED applications. The high structural stabilities of the core–shell PQDs enable the fabrication of PeLEDs through all-solution processes. (3) Crosslinking involves the exchange of conventional OA and OAm with crosslinkable ligands as surface passivation ligands, which suppress defect formation by preventing ligand dissociation through the crosslinking of ligands via light and heat. Crosslinking of surface ligands not only significantly improved the structural stabilities of PQDs, but also enables micro-scale patterning by changing their solubility. High-resolution (~ 1 μm) white PeLEDs are successfully fabricated via the ink-jet printing of green and red emitting PQDs. (4) Metal doping involves the doping of various metal ions with equivalent charge numbers at the A– and B–sites in PQDs, which significantly improves the structural stabilities by changing the B–X bond lengths. Generally, it has been processed by an *in-situ* process during PQDs fabrication. When metal ions are doped into PQDs, both the tolerance and octahedral factors must be considered. The diffusion barrier energy increases when the alkali metal is doped, improving the structural stabilities of the PQDs by slowing their structural degradation.

Despite the in-depth studies on the four strategies for improving the structural stabilities of PQDs, some challenges still remain. The processes for ligand modification and crosslinking methods need to be further simplified and optimized. Furthermore, the development of new ligands with multi-functional groups that are compatible with various ionic materials will further broaden the applications. The issue of insulating and thickness control of inorganic shells (*e.g*., SiO_2_) in core–shell structure should also be considered. Developing of new materials for the shell with high stability and conductivity to replace SiO_2_ could enable a new concept of core–shell PQDs. In addition, the understanding of exact metal doping and defect healing mechanisms remains unclear. Therefore, rather than simply reporting on the enhancement of the opto-electrical properties and structural stabilities of PQDs by metal doping, it is necessary to further elucidate the metal doping and defect healing mechanisms through fundamental analysis. In future applications, PQDs with high structural stabilities are expected to be used as core materials in innovative displays, high efficiency solar cells, high response photodetectors, reliable biosensors, and other applications.

## Data Availability

Not applicable.

## References

[1] Bai PF, Hayes RA, Jin M, Shui L, Yi ZC, Wang L, Zhang X, Zhou G (2014). Prog. Electromagn. Res..

[2] Yuan Q, Wang T, Yu P, Zhang H, Zhang H, Ji W (2021). Org. Electron..

[3] Shinar J, Shinar R (2008). J. Phys. D Appl. Phys..

[4] Kalyani NT, Dhoble S (2015). Renew. Sustain. Energy Rev..

[5] Yang Z, Gao M, Wu W, Yang X, Sun XW, Zhang J, Wang H-C, Liu R-S, Han C-Y, Yang H (2019). Mater. Today.

[6] Tan Z-K, Moghaddam RS, Lai ML, Docampo P, Higler R, Deschler F, Price M, Sadhanala A, Pazos LM, Credgington D (2014). Nat. Nanotechnol..

[7] Yuan S, Liu QW, Tian QS, Jin Y, Wang ZK, Liao LS (2020). Adv. Funct. Mater..

[8] Wang B, Zhou YH, Yuan S, Lou YH, Wang KL, Xia Y, Chen CH, Chen J, Shi YR, Wang ZK (2023). Angew. Chem. Int. Ed..

[9] Kim Y-H, Kim S, Kakekhani A, Park J, Park J, Lee Y-H, Xu H, Nagane S, Wexler RB, Kim D-H (2021). Nat. Photonics.

[10] Kumar S, Marcato T, Krumeich F, Li Y-T, Chiu Y-C, Shih C-J (2022). Nat. Commun..

[11] Kim Y-H, Park J, Kim S, Kim JS, Xu H, Jeong S-H, Hu B, Lee T-W (2022). Nat. Nanotechnol..

[12] Zhang J, Zhang T, Ma Z, Yuan F, Zhou X, Wang H, Liu Z, Qing J, Chen H, Li X (2023). Adv. Mater..

[13] Xiong W, Zou C, Tang W, Xing S, Wang Z, Zhao B, Di D (2023). ACS Energy Lett..

[14] Han JH, Sadhukhan P, Myoung J-M (2023). Appl. Surf. Sci..

[15] Zhang J, Cai B, Zhou X, Yuan F, Yin C, Wang H, Chen H, Ji X, Liang X, Shen C (2023). Adv. Mater..

[16] Wells HL (1893). Z. Anorg. Chem..

[17] Kojima A, Teshima K, Shirai Y, Miyasaka T (2009). J. Am. Chem. Soc..

[18] Liu M, Johnston MB, Snaith HJ (2013). Nature.

[19] Herz LM (2017). ACS Energy Lett..

[20] Sutter-Fella CM, Li Y, Amani M, Ager JW, Toma FM, Yablonovitch E, Sharp ID, Javey A (2016). Nano Lett..

[21] De Wolf S, Holovsky J, Moon S-J, Loper P, Niesen B, Ledinsky M, Haug F-J, Yum J-H, Ballif C (2014). J. Phys. Chem. Lett..

[22] Sutton RJ, Eperon GE, Miranda L, Parrott ES, Kamino BA, Patel JB, Hörantner MT, Johnston MB, Haghighirad AA, Moore DT (2016). Adv. Energy Mater..

[23] Stranks SD, Snaith HJ (2015). Nat. Nanotechnol..

[24] Kulbak M, Cahen D, Hodes G (2015). J. Phys. Chem. Lett..

[25] Correa-Baena J-P, Saliba M, Buonassisi T, Grätzel M, Abate A, Tress W, Hagfeldt A (2017). Science.

[26] Nitika, Dixit SK, Abbas H (2021). J. Mol. Model..

[27] Han TH, Tan S, Xue J, Meng L, Lee JW, Yang Y (2019). Adv. Mater..

[28] Hu X, Zhang X, Liang L, Bao J, Li S, Yang W, Xie Y (2014). Adv. Funct. Mater..

[29] Zhou J, Huang J (2018). Adv. Sci..

[30] Cardenas-Morcoso D, Gualdrón-Reyes ASF, Ferreira Vitoreti AB, García-Tecedor M, Yoon SJ, Solis de la Fuente M, Mora-Seró IN, Gimenez S (2019). J. Phys. Chem. Lett..

[31] Mir WJ, Swarnkar A, Nag A (2019). Nanoscale.

[32] Li C, Guerrero A, Zhong Y, Gräser A, Luna CAM, Köhler J, Bisquert J, Hildner R, Huettner S (2017). Small.

[33] Yassitepe E, Yang Z, Voznyy O, Kim Y, Walters G, Castañeda JA, Kanjanaboos P, Yuan M, Gong X, Fan F (2016). Adv. Funct. Mater..

[34] Meng J, Lan Z, Abdellah M, Yang B, Mossin S, Liang M, Naumova M, Shi Q, Gutierrez Alvarez SL, Liu Y (2020). J. Phys. Chem. Lett..

[35] Seth S, Ahmed T, De A, Samanta A (2019). ACS Energy Lett..

[36] Li X, Zhang K, Li J, Chen J, Wu Y, Liu K, Song J, Zeng H (2018). Adv. Mater. Interfaces.

[37] Bai Z, Zhong H (2015). Sci. Bull..

[38] Cho J, Choi Y-H, O’Loughlin TE, De Jesus L, Banerjee S (2016). Chem. Mater..

[39] Almeida G, Goldoni L, Akkerman Q, Dang Z, Khan AH, Marras S, Moreels I, Manna L (2018). ACS Nano.

[40] Yoo YT, Heo DY, Bae SR, Park J, Lee TW, Jang HW, Ahn SH, Kim SY (2021). Small Methods.

[41] Cho J, Jin H, Sellers DG, Watson DF, Son DH, Banerjee S (2017). J. Mater. Chem. C.

[42] Yang D, Li X, Zeng H (2018). Adv. Mater. Interfaces.

[43] Luo B, Naghadeh SB, Allen AL, Li X, Zhang JZ (2017). Adv. Funct. Mater..

[44] Zhu H, Pan Y, Peng C, Lian H, Lin J (2022). Angew. Chem. Int. Ed..

[45] Wang YK, Singh K, Li JY, Dong Y, Wang XQ, Pina JM, Yu YJ, Sabatini R, Liu Y, Ma D (2022). Adv. Mater..

[46] Zhu H, Tong G, Li J, Xu E, Tao X, Sheng Y, Tang J, Jiang Y (2022). Adv. Mater..

[47] Hassan Y, Park JH, Crawford ML, Sadhanala A, Lee J, Sadighian JC, Mosconi E, Shivanna R, Radicchi E, Jeong M (2021). Nature.

[48] Kumawat NK, Swarnkar A, Nag A, Kabra D (2018). J. Phys. Chem. C.

[49] Bi C, Kershaw SV, Rogach AL, Tian J (2019). Adv. Funct. Mater..

[50] Kim JS, Heo J-M, Park G-S, Woo S-J, Cho C, Yun HJ, Kim D-H, Park J, Lee S-C, Park S-H (2022). Nature.

[51] Li G, Huang J, Zhu H, Li Y, Tang J-X, Jiang Y (2018). Chem. Mater..

[52] Kim Y-H, Lee G-H, Kim Y-T, Wolf C, Yun HJ, Kwon W, Park CG, Lee T-W (2017). Nano Energy.

[53] Choi JW, Woo HC, Huang X, Jung W-G, Kim B-J, Jeon S-W, Yim S-Y, Lee J-S, Lee C-L (2018). Nanoscale.

[54] Pan J, Quan LN, Zhao Y, Peng W, Murali B, Sarmah SP, Yuan M, Sinatra L, Alyami NM, Liu J (2016). Adv. Mater..

[55] Pan J, Sarmah SP, Murali B, Dursun I, Peng W, Parida MR, Liu J, Sinatra L, Alyami N, Zhao C (2015). J. Phys. Chem. Lett..

[56] Lee H, Trinh CK, So MG, Lee C-L (2022). Nanoscale.

[57] Wei Y, Cheng Z, Lin J (2019). Chem. Soc. Rev..

[58] Raja SN, Bekenstein Y, Koc MA, Fischer S, Zhang D, Lin L, Ritchie RO, Yang P, Alivisatos AP, Appl ACS (2016). Mater. Interfaces.

[59] Li ZJ, Hofman E, Li J, Davis AH, Tung CH, Wu LZ, Zheng W (2018). Adv. Funct. Mater..

[60] Zhong Q, Cao M, Hu H, Yang D, Chen M, Li P, Wu L, Zhang Q (2018). ACS Nano.

[61] Gao F, Yang W, Liu X, Li Y, Liu W, Xu H, Liu Y (2021). Chem. Eng. J..

[62] Park J, Jang KY, Lee SH, Kim D-H, Cho S-H, Lee T-W (2023). Chem. Mater..

[63] Kim JY, Kim BG, Kim M, Jang W, Wang DH (2021). J. Alloys Compd..

[64] Liu Y, Li F, Liu Q, Xia Z (2018). Chem. Mater..

[65] Trinh CK, Lee H, So MG, Lee C-L, Appl ACS (2021). Mater. Interfaces.

[66] Li G, Rivarola FWR, Davis NJ, Bai S, Jellicoe TC, de la Peña F, Hou S, Ducati C, Gao F, Friend RH (2016). Adv. Mater..

[67] Tang J, Kemp KW, Hoogland S, Jeong KS, Liu H, Levina L, Furukawa M, Wang X, Debnath R, Cha D (2011). Nat. Mater..

[68] Wei Y, Li X, Chen Y, Cheng Z, Xiao H, Li X, Ding J, Lin J (2020). J. Phys. Chem. Lett..

[69] Fan Y, Fang Y, Ma L (2014). Colloids Surf. B Biointerfaces.

[70] Erinç H, Isler I (2019). Acta Aliment..

[71] Lee H, Jeong JW, So MG, Jung GY, Lee CL (2021). Adv. Mater..

[72] Zhou Y, Chen J, Bakr OM, Sun H-T (2018). Chem. Mater..

[73] Travis W, Glover E, Bronstein H, Scanlon D, Palgrave R (2016). Chem. Sci..

[74] Li W, Wang Z, Deschler F, Gao S, Friend RH, Cheetham AK (2017). Nat. Rev. Mater..

[75] Bi C, Wang S, Li Q, Kershaw SV, Tian J, Rogach AL (2019). J. Phys. Chem. Lett..

[76] Protesescu L, Yakunin S, Bodnarchuk MI, Krieg F, Caputo R, Hendon CH, Yang RX, Walsh A, Kovalenko MV (2015). Nano Lett..

[77] Yao J-S, Ge J, Han B-N, Wang K-H, Yao H-B, Yu H-L, Li J-H, Zhu B-S, Song J-Z, Chen C (2018). J. Am. Chem. Soc..

[78] Lu M, Zhang X, Zhang Y, Guo J, Shen X, Yu WW, Rogach AL (2018). Adv. Mater..

[79] Das S, De A, Samanta A (2020). J. Phys. Chem. Lett..

[80] Li S, Shi Z, Zhang F, Wang L, Ma Z, Yang D, Yao Z, Wu D, Xu T-T, Tian Y (2019). Chem. Mater..

[81] Hoang MT, Pannu AS, Tang C, Yang Y, Pham ND, Gui K, Wang X, Yambem S, Sonar P, Du A (2020). Adv. Opt. Mater..

[82] Amgar D, Binyamin T, Uvarov V, Etgar L (2018). Nanoscale.

[83] Van Den Stam W, Geuchies JJ, Altantzis T, Van Den Bos KH, Meeldijk JD, Van Aert S, Bals S, Vanmaekelbergh D, de Mello Donega C (2017). J. Am. Chem. Soc..

[84] Zeng F, Tan Y, Hu W, Tang X, Zhang X, Yin H (2022). J. Lumin..

[85] Begum R, Parida MR, Abdelhady AL, Murali B, Alyami NM, Ahmed GH, Hedhili MN, Bakr OM, Mohammed OF (2017). J. Am. Chem. Soc..

[86] Wang Q, Wang X, Yang Z, Zhou N, Deng Y, Zhao J, Xiao X, Rudd P, Moran A, Yan Y (2019). Nat. Commun..

[87] Lu C-H, Biesold-McGee GV, Liu Y, Kang Z, Lin Z (2020). Chem. Soc. Rev..

[88] Swarnkar A, Mir WJ, Nag A (2018). ACS Energy Lett..

[89] Cao J, Tao SX, Bobbert PA, Wong CP, Zhao N (2018). Adv. Mater..

[90] Liu M, Zhong G, Yin Y, Miao J, Li K, Wang C, Xu X, Shen C, Meng H (2017). Adv. Sci..

[91] He J, Vasenko AS, Long R, Prezhdo OV (2018). J. Phys. Chem. Lett..

